# Improved Detection of Circulating Tumor Cells in Metastatic Colorectal Cancer by the Combination of the CellSearch^®^ System and the AdnaTest^®^

**DOI:** 10.1371/journal.pone.0155126

**Published:** 2016-05-16

**Authors:** Tobias M. Gorges, Alexander Stein, Julia Quidde, Siegfried Hauch, Katharina Röck, Sabine Riethdorf, Simon A. Joosse, Klaus Pantel

**Affiliations:** 1 Department of Tumor Biology, University Medical Center Hamburg-Eppendorf, Hamburg, Germany; 2 Department of Internal Medicine II and Clinic (Oncology Center), University Medical Center Hamburg-Eppendorf, Hamburg, Germany; 3 AdnaGen GmbH, Langenhagen, Germany; Chang Gung University, TAIWAN

## Abstract

Colorectal cancer (CRC) is one of the major causes of cancer-related death and reliable blood-based prognostic biomarkers are urgently needed. The enumeration and molecular characterization of circulating tumor cells (CTCs) has gained increasing interest in clinical practice. CTC detection by CellSearch^®^ has already been correlated to an unfavorable outcome in metastatic CRC. However, the CTC detection rate in mCRC disease is low compared to other tumor entities. Thus, the use of alternative (or supplementary) assays might help to itemize the prognostic use of CTCs as blood-based biomarkers. In this study, blood samples from 47 mCRC patients were screened for CTCs using the FDA-cleared CellSearch^®^ technology and / or the AdnaTest^®^. 38 samples could be processed in parallel. We demonstrate that a combined analysis of CellSearch^®^ and the AdnaTest^®^ leads to an improved detection of CTCs in our mCRC patient cohort (positivity rate CellSearch^®^ 33%, AdnaTest^®^ 30%, combined 50%). While CTCs detected with the CellSearch^®^ system were significantly associated with progression-free survival (p = 0.046), a significant correlation regarding overall survival could be only seen when both assays were combined (p = 0.013). These findings could help to establish improved tools to detect CTCs as on-treatment biomarkers for clinical routine in future studies.

## Introduction

Cancer-related death is usually caused by the outgrowth of aggressive cancer cells at new locations in the body (metastasis formation) that have been disseminated from the primary tumors. Colorectal cancer (CRC) is one of the most commonly diagnosed malignancies and one of the leading causes of cancer related deaths [1]. Approximately one quarter of patients with CRC exhibit metastases (mCRC) at the time of diagnosis (synchronous disease) and further patients will develop metastases during the course of their disease, resulting in the relatively high mortality rate associated with CRC [2].

Different prognostic markers can currently be used in mCRC, e.g., analyses of white blood cells, lactate dehydrogenase amount, performance status, localization of primary tumor and metastases, molecular markers (e.g., mutations in the BRAF gene), or advanced integrative clustering) [3–7]. Besides, tumor growth dynamics have been shown to offer on-treatment information about future prognosis [8, 9].

Enumeration and molecular characterization of tumor cells captured from a minimally invasive blood test (circulating tumor cells, CTCs) is increasingly used in clinical practice for disease monitoring and discovering prognostic relevance [10]. CTCs are easy to obtain by less-invasive peripheral blood sampling allowing a continuous “real-time monitoring” of tumor progression. So far, the CellSearch^®^ system is the only approach which has been cleared by the U.S. American FDA (Food and Drug Administration) for CTC detection in metastatic breast, colon, and prostate cancer [11–13]. Detection of CTCs using CellSearch^®^ has recently been correlated to an unfavorable outcome in mCRC [14]. However, studies performed in mCRC patients using CellSearch^®^ demonstrated a much lower yield of CTCs in this tumor type compared with breast or prostate cancer [11–13]. It is expected that “only” 30–40% of patients with mCRC harbor 3 or more CTCs per 7.5 ml of blood [15]. Therefore, the use of other (or supplementary) assays might improve our understanding of the mechanisms of cancer biology and itemize the use of CTCs as cancer biomarkers in mCRC. For example, significant discordance between CellSearch^®^ and the AdnaTest^®^ in the detection of CTCs from mCRC patients has already been observed [16]. The CellSearch^®^ system uses immunostaining and defines CTCs as cells expressing both EpCAM and pankeratins and not expressing CD45, as well as having a nucleus stained with DAPI (4′,6-diamidino-2-phenylindole). In contrast, the AdnaTest^®^ uses an RT-PCR platform targeting three different transcripts (*EpCAM*, *EGFR*, *CEA*) to identify the tumor cells within the EpCAM-enriched cell fraction.

A combined analysis of both assays could therefore improve the sensitivity of CTC detection by applying two methodological approaches (immunocytochemistry and RT-PCR) and increasing the range of CTC markers. This is an important advantage in view of the well-known phenotypic heterogeneity of CTCs [10]. The purpose of this study was to analyze the clinical relevance of CTCs by comparing and combining two different assays for CTC detection in a small cohort of mCRC patients (n = 47). For this aim, the AdnaTest^®^ and the CellSearch^®^ system were employed in parallel. Our data shows that a combined analysis of both assays leads to increased detection rates of CTCs with additional prognostic information. These findings could help to establish new diagnostic tools to use of CTCs as on-treatment biomarkers for clinical routine in future studies.

## Material and Methods

### Patient series

Consecutive patients scheduled for palliative chemotherapy for CRC from the out-patient clinic from the Department of Oncology and Hematology at the University Medical Center Hamburg-Eppendorf were recruited. Patient characteristics are summarized in Table 1. The majority of patients were already extensively pretreated, receiving the 3^rd^ (median) line treatment (range: 1–8). Overall, more than 80% of patients received fluoropyrimidines, oxaliplatin, irinotecan, and bevacizumab and about 40% of patients EGFR antibodies. The demographics and patterns of metastasis were as expected, although the overall population was slightly younger than the median metastatic CRC population, likely related to the university hospital background. The study was carried out in accordance with the World Medical Association Declaration of Helsinki and the guidelines for experimentation with humans by the Chambers of Physicians of the State of Hamburg (“Hamburger Ärztekammer”). The experimental protocol was approved (Approval No. PVN-3779) by the Ethics Committee of the Chambers of Physicians of the State of Hamburg (“Hamburger Ärztekammer”). All participants gave written informed consent before the study began. In total, blood samples (5 ml and 7.5 ml) from 47 patients were collected into AdnaCollect^®^ blood collection tubes (AdnaGen^®^) or CellSave^®^ preservation tubes (Janssen Diagnostics), and processed within 24 h (AdnaTest^®^) or 96 h (CellSearch^®^) according to the guidelines of the vendors.

**Table 1:** Patient characteristics.

**Patient characteristics at first diagnosis (n = 47)**	
Age (years)	Median 56 (range 37–79)
Gender	male (n = 29) / female (n = 15) / n.d. (n = 3)
T stage	I (n = 0) / II (n = 2) / III (n = 20) / IV (n = 9) / n.d. (n = 16)
N stage	0 (n = 8) / I (n = 13) / II (n = 11) / n.d. (n = 16)
M stage	0 (n = 11) / 1 (n = 28) / n.d. (n = 8)
*KRAS* staus	Wild type (n = 25) / Mutated (n = 21)
**Patient characteristics at blood withdrawal**	
Liver metastases	Positive (n = 39) / Negative (n = 7)
Lung metastases	Positive (n = 26) / Negative (n = 20)
Lymph node metastases	Positive (n = 10) / Negative (n = 36)
Bone metastases	Positive (n = 4) / Negative (n = 42)
Therapy line	1^st^ (n = 4) / 2^nd^ (n = 9) / 3^rd^ (n = 9) / 4^th^ (n = 6) / 5^th^ (n = 9) / 6^th^ (n = 4) / 7^th^ (n = 2) / 8^th^ (n = 1)

Patient characteristics at time point of diagnosis and blood withdrawal.

### AdnaTest^®^

For the enrichment and analysis of circulating tumor cells (CTC) the AdnaTest ColonCancerSelect and the AdnaTest ColonCancerDetect, (AdnaGen GmbH, Langenhagen) were used to prepare mRNA, followed by a RT-PCR for a later multiplex PCR according to the manufacturer’s instructions [16]. All required information regarding sample processing can be found on the webpage http://www.adnagen.com. Briefly, 5 ml of blood was taken for an enrichment of CTC by using antibody-coated magnetic particles consisting of a mixture of antibodies against different EpCAM epitopes The enriched cells were subsequently lysed and mRNA was purified by means of oligo-dT beads contained in the kit followed by reverse transcription (Sensiscript, Qiagen, Hilden). The resulting cDNA was processed in a multiplex PCR for tumor-associated transcripts (*epidermal growth factor receptor* (*EGFR*), *carcinoembryonic antigen* (*CEA*) and *EpCAM*) as well as *Actin* as housekeeping control. PCR was performed using the HotStarTaq Master Mix (QIAGEN GmbH, Hilden, Germany). Visualization of the PCR fragments was carried out with a 2100 Bioanalyzer using the DNA1000 assay (Agilent Technologies, Waldbronn, Germany). CTCs were positively identified if at least one of the multiplex PCR markers was detected.

### CellSearch^®^

For isolation of CTCs using CellSearch^®^, CTC detection was performed as described elsewhere [17]. The criteria for an event to be defined as CTC were: a round to oval morphology, a visible nucleus (DAPI-positive), and a positive staining pattern for an epithelial specific cell (Keratin-positive and CD45-negative). For EGFR determination on CTCs, the CellSearch^®^ Tumor Phenotyping Reagent EGFR was applied in the fourth channel of the system [18].

### Statistical analysis

In order to compare the results of the CellSearch^®^ system and the AdnaTest^®^ experiments, CTC counts from CellSearch^®^ data were transformed to positive (≥3 CTCs) or negative (<3 CTCs) since ≥3 CTCs / 7.5 ml of blood have been associated to poor clinical outcome [13]. CTC status (positive / negative) and correlation with metastasis location was tested with 2x2 Fisher’s exact test [19] and corrected for multiple testing. McNemar’s test with Yate’s correction for continuity was performed to find the agreement between the two methods. Progression-free and overall survival (PFS and OS) estimates for both methods were calculated by Kaplan-Meyer curves and compared by log-rank test [20].

## Results

### Clinical sample analysis using the AdnaTest^®^ and CellSearch^®^

**Fig 1:**
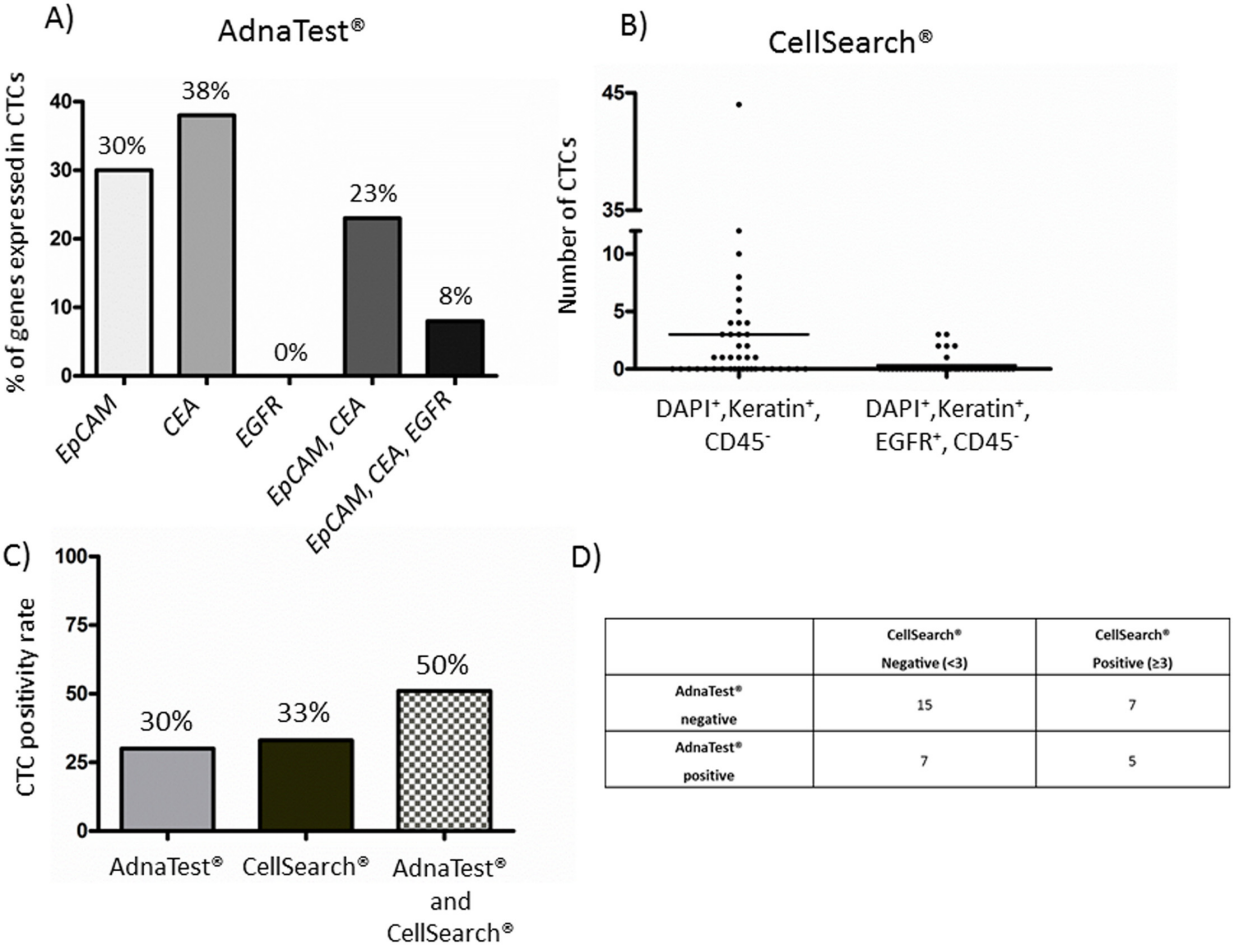
(A) Summarized marker analyses of CTCs detected with the AdnaTest^®^. CTCs were positively identified if at least one of the multiplex PCR markers (*EpCAM*, *CEA*, or *EGFR*) was detected. (B) CTC enumeration by CellSearch^®^ including EGFR determination. (C) CTC positivity rate of the AdnaTest^®^, CellSearch^®^, or both assay in combination. (D) Comparison between the AdnaTest^®^ and the CellSearch^®^ system.

Using the AdnaTest^®^, 13 out of 43 (30%) analyzed patients were positive for CTCs. CTCs were positively identified if at least one of the multiplex PCR markers was detected. CTCs from 8 patients exhibited positive signals for *EpCAM*. Four out of those patients additionally showed signals for *CEA*, whereas one patient was positive for *EpCAM*, *CEA*, and *EGFR*. Five patients showed exclusively signals for *CEA* without expression signals for any other marker (Fig 1A). For CellSearch^®^ analyses, only patients with ≥3 CTCs were classified as “CTC-positive” because this cut-off was correlated to an unfavorable outcome in mCRC [13, 21]. Fourteen out of 42 (33%) analyzed samples were positive for CTCs (range: 3–44 cells). EGFR-positive cells (moderate to strong expression) were found in six out of those 14 patients (range from 1–4 EGFR-positive cells within the CTC-positive cohort) (Fig 1B). In this study, 38 clinical blood samples could be analyzed in parallel. Combining the AdnaTest^®^ and CellSearch^®^ 19 out of 38 (50%) analyzed samples became positive for CTCs (Fig 1C). Within this group, *EGFR*/EGFR signals did not correlate since only one patient was *EGFR*-positive with the AdnaTest^®^, but had EGFR-negative CTCs with CellSearch^®^ (patient 42). A detailed summary of the CTC analyses is listed in Table 2. Fifteen samples were CTC-negative in both CTC assays and 5 patients were CTC-positive in both assays. Seven samples were positive in CellSearch^®^ and negative for the AdnaTest^®^, whereas seven cases were exclusively positive for AdnaTest^®^. The results of both assays did not correlate significantly (Cohen's kappa = 0.1066, p = 0.51) (Fig 1D).

**Table 2:** Detailed patient characteristics and CTC results.

	Status at first diagnoses			Current status at blood withdrawal								
Patient	T	N	M	Liver	Lung	Lymphnode	Bone	AdnaTest^®^ (transcripts)	CellSearch^®^ (CTC count)	CTCs positive for EGFR (CellSearch^®^)	Line of treatment	Remission status
1	3	2	1	1	1	0	0	0	4	0	5	PD
2	4	2	0	1	1	0	0	EpCAM/CEA	1	0	5	SD
3	2	2	1	1	0	0	0	EpCAM/CEA	0	0	1	SD
4	-	-	-	0	0	0	0	EpCAM	1	0	4	SD
5	3	1	1	1	1	1	0	0	0	0	4	SD
6	4	0	0	1	1	0	0	0	-	-	2	SD
7	-	-	-	1	0	1	0	0	-	-	2	SD
8	-	-	1	1	0	0	0	0	6	0	5	PD
9	3	0	1	1	1	0	0	0	1	0	4	SD
10	-	-	1	1	0	0	0	CEA	0	0	3	PD
11	3	0	1	1	0	0	0	0	0	0	2	PD
12	3	0	0	1	0	0	0	EpCAM/CEA	8	2	7	PD
13	-	-	-	1	1	0	0	0	0	0	4	PD
14	-	-	1	1	1	0	0	0	1	0	2	SD
15	-	-	1	1	1	0	0	CEA	4	0	3	SD
16	-	-	-	-	-	-	-	0	0	0	-	-
17	4	1	1	1	0	1	0	0	0	0	4	SD
18	3	0	0	1	1	0	0	0	10	3	5	PD
19	3	1	0	1	1	0	0	CEA	-	-	5	SD
20	3	1	1	1	1	1	0	0	0	0	5	SD
21	3	1	0	1	1	0	0	EpCAM	5	3	8	SD
22	3	1	0	1	0	0	0	-	0	0	2	-
23	3	1	1	0	1	0	0	0	0	0	3	SD
24	3	2	1	0	0	1	0	0	0	0	2	-
25	2	2	-	1	1	0	0	0	0	0	5	PD
26	3	0	-	1	0	1	0	-	1	0	3	PD
27	-	-	1	1	1	0	0	0	-	-	2	PD
28	4	1	1	1	0	1	0	0	0	0	1	PD
29	-	-	1	1	1	0	1	0	-	-	3	PD
30	3	2	1	1	0	0	0	0	12	0	3	PD
31	-	-	1	1	0	0	0	-	3	1	-	PD
32	-	1	1	1	0	1	0	0	7	0	1	SD
33	3	1	0	0	1	0	0	CEA	1	0	4	SD
34	3	0	1	0	1	0	1	0	0	0	6	SD
35	4	1	1	1	1	0	0	0	3	2	3	SD
36	4	1	0	1	1	0	0	EpCAM	0	0	2	SD
37	4	2	1	1	1	0	0	0	0	0	5	PD
38	-	-	-	1	0	0	0	0	0	0	3	PD
39	3	2	0	1	1	0	0	CEA	2	0	7	RM
40	4	2	1	1	0	0	0	0	2	0	5	PD
41	-	-	1	1	1	0	0	EpCAM	3	2	6	PD
42	3	2	1	1	1	0	0	EpCAM/CEA/EGFR	44	0	1	PD
43	3	2	1	1	0	0	1	0	0	0	3	SD
44	-	-	1	0	0	0	0	0	0	0	2	SD
45	-	-	-	0	1	1	1	0	4	0	6	-
46	4	0	0	1	1	0	0	-	3	0	-	SD
47	3	1	1	1	0	1	0	-	0	0	6	-

Table 2 discloses detailed patient characteristics of 47 metastatic colorectal cancer patients and their corresponding CTC results (AdnaTest^®^ or CellSearch^®^). Remission status after blood withdrawal is indicated as stable disease (SD), progressive disease (PD), or remission (RM). Missing data points are indicated as (-).

### Correlation of CTC findings to metastatic site and therapy

Using the AdnaTest^®^, no correlation was found regarding CTC-positivity and the location of metastases (p>0.05). Similar findings were observed when using CellSearch^®^ (p>0.05). Combining both assays also did not result in a significant correlation between metastasis location and CTC status (p>0.05). CTC status was not correlated with having one or multiple metastases (p>0.05) in any of the CTC detection methods. Interestingly, CTC positivity rate was associated with a higher line of therapy, median 2^nd^ line in case of CTC negativity compared to median 4^th^ line in case of CTC positivity.

### Correlation of CTC findings to clinical outcome

**Fig 2:**
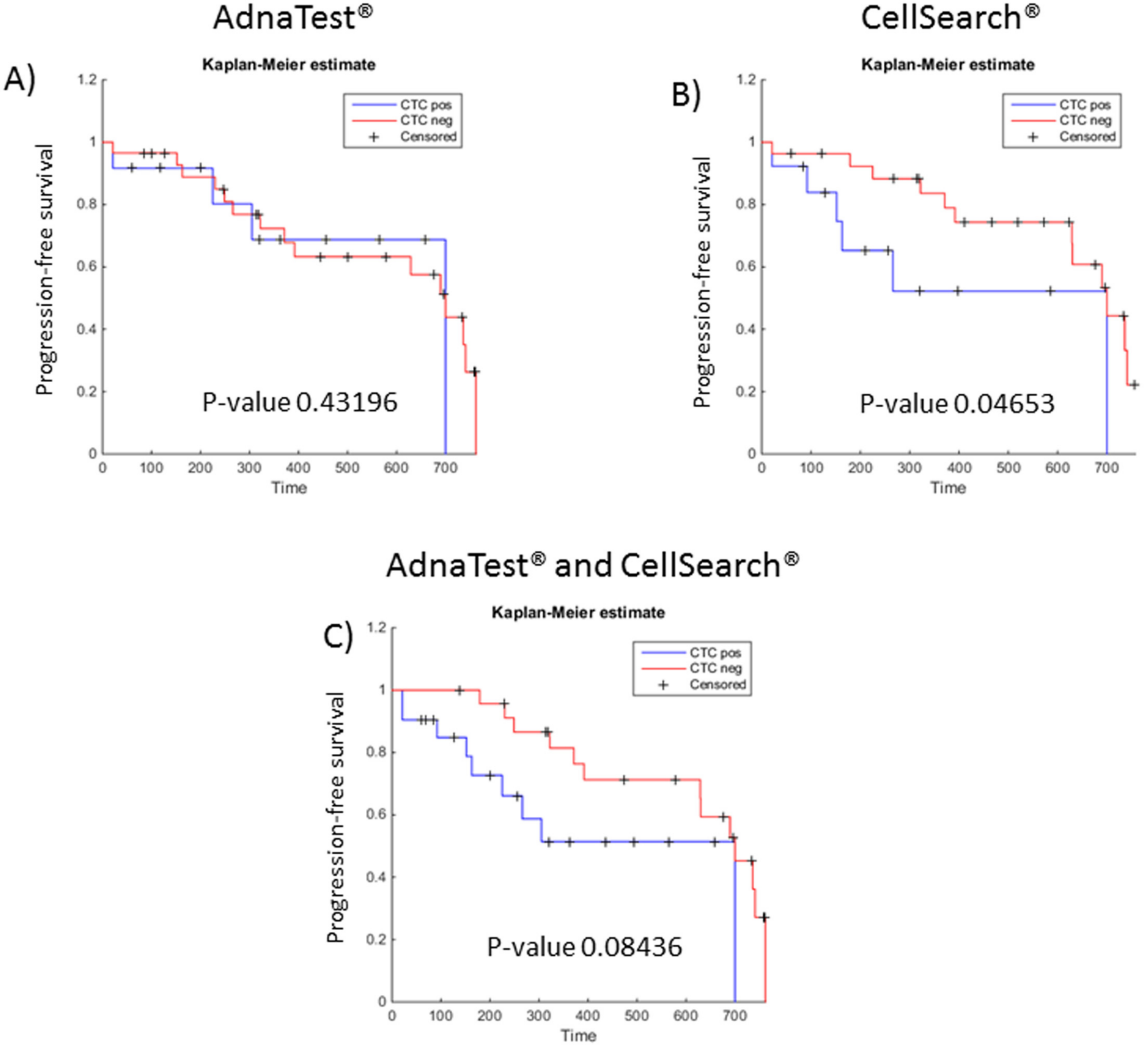
Kaplan-Meier curves for progression-free survival according to the CTC status using the AdnaTest^®^ (A), CellSearch^®^ (B), or both assay in combination (C).

Kaplan-Meyer curves and log-rank statistic for CTC-negative and CTC-positive cases revealed no significant correlation regarding progression-free survival (PFS) when using the AdnaTest^®^ (p = 0.43) (Fig 2A). For CellSearch^®^ a significant association between PFS and CTC status was observed (p = 0.0467) (Fig 2B). A combination of both assays again did not show a significant correlation to PFS but revealed a trend (p = 0.084) (Fig 2C).

**Fig 3:**
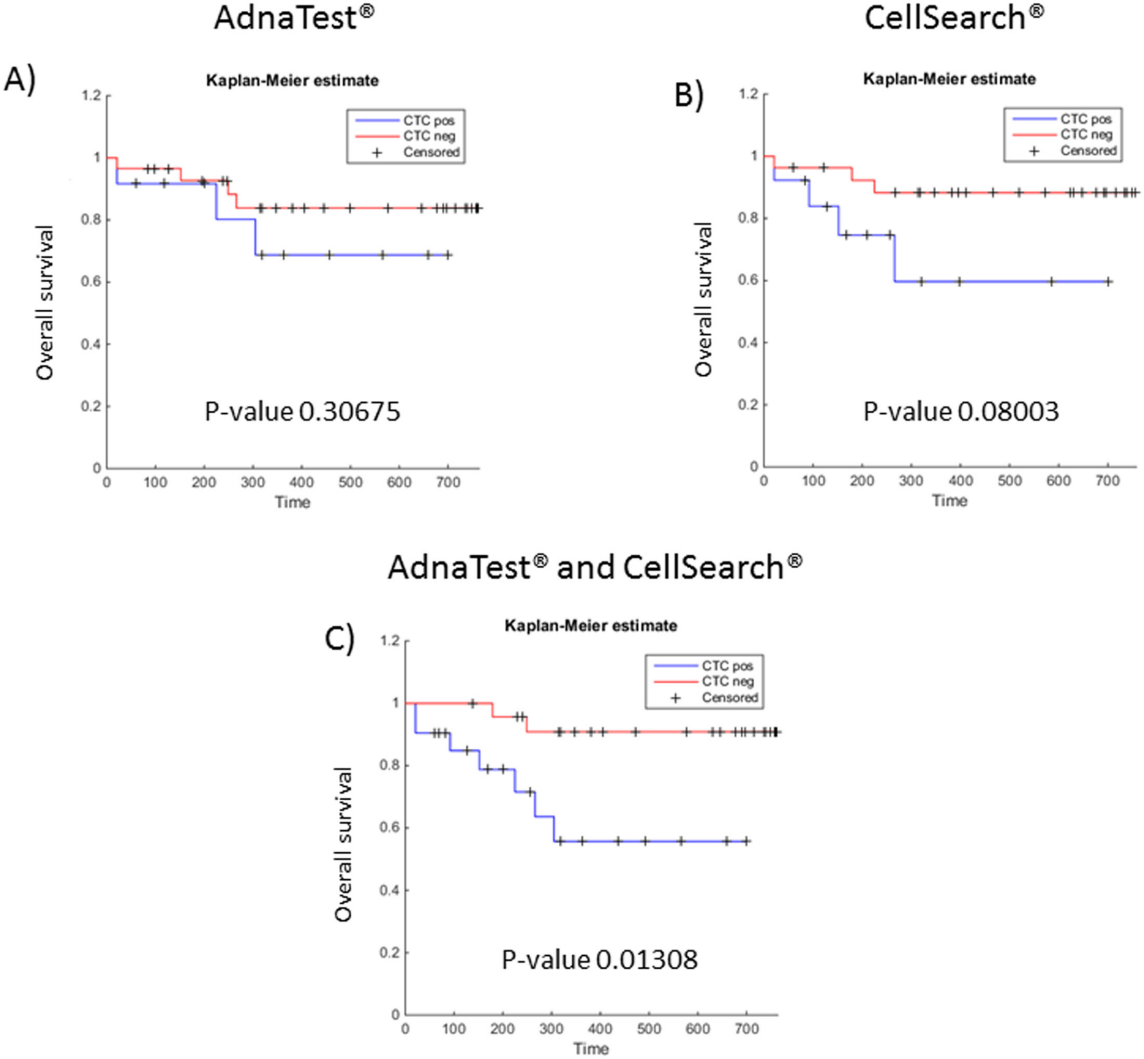
Kaplan-Meier curves for overall survival according to the CTC status using the AdnaTest^®^ (A), CellSearch^®^ (B), or both assay in combination (C).

We also tested whether the presence of CTCs by the AdnaTest^®^, CellSearch^®^ or the combined results were associated with reduced overall survival (OS) in our mCRC patient cohort. Using Kaplan-Meier analysis a significant correlation could be only seen when both assays were combined (p = 0.013), while the individual assays alone provided no significant prognostic information (AdnaTest^®^: p = 0.31, CellSearch^®^: p = 0.080) (Fig 3A–3C).

## Discussion

Colorectal cancer is one of the major causes of cancer-related death and reliable blood-based biomarkers are urgently needed to improve upfront treatment selection and modifications during treatment. To date, the most widely used blood-based marker in CRC is CEA to get prognostic information at baseline and predictive information during treatment [22–24]. Despite the wide spread use of CEA as a blood biomarker it is neither disease specific, influenced by other factors, and not as reliable as other more recent blood based biomarkers (e.g. CTCs) [25].

OS is the most reliable endpoint in clinical studies and the detection of CTCs has already been shown to have a prognostic impact in many tumor entities, including mCRC [11, 12, 21, 26–28]. In our cohort, we could observe higher CTC detection rates and better prognostic information concerning OS when combing two different CTC detection systems—the FDA-cleared CellSearch^®^ technology and the AdnaTest^®^. The prognosis of PFS significantly correlated with CTC detection when using CellSearch^®^ alone (p-value 0.046 vs. 0.43 when using the AdnaTest^®^ and 0.084 for combined analyses of both assays). However, PFS by definition refers to the date on which progression is detected and furthermore depends on radiological evaluation by a clinician, making PFS a more unreliable endpoint.

Significant discordances between CellSearch^®^ and the AdnaTest^®^ in the detection of CTCs were already published [16, 29, 30]. However, none of these studies correlated the incidence of CTCs to clinical outcome. In the present study, a combination of the CellSearch^®^ system and the AdnaTest^®^ increased the detection rate from 30% to 50% (30% when using the AdnaTest^®^; 33% when using CellSearch^®^ (≥3 CTCs); 50% if both assays were combined). CTC positivity rate seems to be associated with advanced line of treatment (Table 2), which argues in favor of the assumption that higher CTC counts enable patients to acquire resistance to systemic therapies. In view of the marked heterogeneity of CTCs in CRC [31], the presence of more CTCs should increase the chance to harbor resistant clones.

Although we could clearly demonstrate that both methods in combination improve the positivity rate for CTCs, 50% of the patients were still negative despite the presence of overt metastases. Besides technical limitations of the assays (see next paragraph) there might be an interesting biology behind the observation that CTC levels in CRC are lower than in breast cancer. One might argue that tumor cell dissemination in CRC is less pronounced, which would also explain why CRC patients with liver metastases can be cured by surgery in approximately 20%, while such cure is rarely achieved by the same treatment in breast cancer.

Both assays used in our studies rely on the expression of epithelial cell surface markers and are, therefore, likely to miss CTC populations which have undergone a complete epithelial-to-mesenchymal transition (EMT) [32–34]. However, recent work has been shown that cancer cells with an intermediate phenotype are probably the most aggressive ones able to disseminate and outgrow at distant sites because of their high plasticity [35, 36]. The CellSearch^®^ system and the AdnaTest^®^ are both able to detect EMT-associated CTCs [37, 38]. Thus, the CTC assays used in the present investigation target epithelial and intermediate CTCs, while pure mesenchymal CTCs (lacking any expression of epithelial markers) are probably lost. The detection of pure mesenchymal CTCs is anyway very difficult because even if label-free (i.e., EpCAM-independent) capture systems are used (e.g., filters) epithelial markers such as keratins are usually applied for the detection of CTCs because of the lack of mesenchymal markers that are not expressed on the surrounding blood cells. In this context, plastin-3, an actin-bundling protein not downregulated during EMT on CRC and breast cancer cells—and not expressed on leukocytes—might be a major advance [33, 34].

Cell-free nucleic acids (e.g., cell-free tumor DNA or miRNAs) are also currently discussed to be suitable as novel blood-based biomarkers [39]. Changes in their concentration as well as DNA alterations were already shown to be used for diagnostic, treatment monitoring, predictive, or prognostic purposes [40–43]. However, the majority of ctDNA is derived from apoptotic tumor cells, while CTC analysis has the advantage to study viable tumor cells, which may also improve our current understanding of tumor cell dissemination in cancer patients. Furthermore, so far no standardized assay / protocol for the detection of cell-free nucleic acids or miRNAs could be established in mCRC disease and different approaches for the discovery of those biomarkers are used in the laboratories all over the world. Just recently, a standardized workflow strategy was suggested for the normalization of miRNA expression data as a novel starting point for standardized procedures to allow data comparison across different laboratories [44]. The development of standardized assys that can be used for companion diagnostics is the focus of the newly established EU-IMI consortium called CANCER-ID (www.Cancer-id.eu).

Lots of debate is ongoing whether CTCs can be used as liquid biopsies guiding individualized treatment decisions [10]. Using the AdnaTest^®^ and CellSearch^®^ we were able to detect relevant therapeutic targets such as *EGFR*/EGFR. Signals for *EGFR*/EGFR on CTCs did not correlate in our study between the AdnaTest^®^ and CellSearch^®^ (Table 2), which is probably due to the different CTC populations captured with our assays. This shows again the complementarity of both assays.

Summing up, we could show that a combination of different CTC assays increases the appearance of CTCs in an unselected group of mCRC patients. A significant correlation to OS could be only seen when both assays were combined (p = 0.013). These findings could help to establish CTCs as on-treatment biomarkers for clinical routine in future studies.

## References

[1] Malvezzi M, Bertuccio P, Levi F, La Vecchia C, Negri E. European cancer mortality predictions for the year 2014. Annals of oncology: official journal of the European Society for Medical Oncology / ESMO. 2014;25(8):1650–6. 10.1093/annonc/mdu138 . 24759568

[2] Siegel R, Desantis C, Jemal A. Colorectal cancer statistics, 2014. CA Cancer J Clin. 2014;64(2):104–17. 10.3322/caac.21220 . 24639052

[3] Kohne CH, Cunningham D, Di CF, Glimelius B, Blijham G, Aranda E, et al Clinical determinants of survival in patients with 5-fluorouracil-based treatment for metastatic colorectal cancer: results of a multivariate analysis of 3825 patients. Ann Oncol. 2002;13(2):308–17. . 11886010 10.1093/annonc/mdf034

[4] Sadanandam A, Lyssiotis CA, Homicsko K, Collisson EA, Gibb WJ, Wullschleger S, et al A colorectal cancer classification system that associates cellular phenotype and responses to therapy. Nat Med. 2013;19(5):619–25. Epub 2013/04/16. nm.3175 [pii] 10.1038/nm.3175 . 23584089 PMC3774607

[5] Zhang B, Wang J, Wang X, Zhu J, Liu Q, Shi Z, et al Proteogenomic characterization of human colon and rectal cancer. Nature. 2014;513(7518):382–7. Epub 2014/07/22. 10.1038/nature13438 . 25043054 PMC4249766

[6] Missiaglia E, Jacobs B, D'Ario G, Di Narzo AF, Soneson C, Budinska E, et al Distal and proximal colon cancers differ in terms of molecular, pathological, and clinical features. Ann Oncol. 2014;25(10):1995–2001. Epub 2014/07/25. 10.1093/annonc/mdu275 . 25057166

[7] Venderbosch S, Nagtegaal ID, Maughan TS, Smith CG, Cheadle JP, Fisher D, et al Mismatch Repair Status and BRAF Mutation Status in Metastatic Colorectal Cancer Patients: A Pooled Analysis of the CAIRO, CAIRO2, COIN, and FOCUS Studies. Clin Cancer Res. 2014;20(20):5322–30. Epub 2014/08/21. 10.1158/1078-0432.CCR-14-0332 25139339 PMC4201568

[8] Piessevaux H, Buyse M, Schlichting M, Van Cutsem E, Bokemeyer C, Heeger S, et al Use of early tumor shrinkage to predict long-term outcome in metastatic colorectal cancer treated with cetuximab. J Clin Oncol. 2013;31(30):3764–75. Epub 2013/09/18. JCO.2012.42.8532 [pii] 10.1200/JCO.2012.42.8532 . 24043732

[9] Mansmann U, Sartorius U. Deepness of response: A quantitative analysis of its impact on post-progression survival time after first-line treatment in patients with mCRC. J Clin Oncol. 2013;30(suppl 34):abstr 427.

[10] Alix-Panabieres C, Pantel K. Challenges in circulating tumour cell research. Nature reviews Cancer. 2014;14(9):623–31. Epub 2014/08/27. 10.1038/nrc3820 . 25154812

[11] Cristofanilli M, Budd GT, Ellis MJ, Stopeck A, Matera J, Miller MC, et al Circulating tumor cells, disease progression, and survival in metastatic breast cancer. N Engl J Med. 2004;351(8):781–91. Epub 2004/08/20. 10.1056/NEJMoa040766 351/8/781 [pii]. . 15317891

[12] de Bono JS, Scher HI, Montgomery RB, Parker C, Miller MC, Tissing H, et al Circulating tumor cells predict survival benefit from treatment in metastatic castration-resistant prostate cancer. Clinical cancer research: an official journal of the American Association for Cancer Research. 2008;14(19):6302–9. Epub 2008/10/03. 10.1158/1078-0432.ccr-08-0872 . 18829513

[13] Cohen SJ, Punt CJ, Iannotti N, Saidman BH, Sabbath KD, Gabrail NY, et al Relationship of circulating tumor cells to tumor response, progression-free survival, and overall survival in patients with metastatic colorectal cancer. Journal of clinical oncology: official journal of the American Society of Clinical Oncology. 2008;26(19):3213–21. Epub 2008/07/02. 10.1200/jco.2007.15.8923 . 18591556

[14] Huang X, Gao P, Song Y, Sun J, Chen X, Zhao J, et al Meta-analysis of the prognostic value of circulating tumor cells detected with the CellSearch System in colorectal cancer. BMC cancer. 2015;15:202 Epub 2015/04/17. 10.1186/s12885-015-1218-9 25880692 PMC4389311

[15] Negin BP, Cohen SJ. Circulating tumor cells in colorectal cancer: past, present, and future challenges. Current treatment options in oncology. 2010;11(1–2):1–13. Epub 2010/02/10. 10.1007/s11864-010-0115-3 . 20143276

[16] Raimondi C, Nicolazzo C, Gradilone A, Giannini G, De Falco E, Chimenti I, et al Circulating tumor cells: exploring intratumor heterogeneity of colorectal cancer. Cancer biology & therapy. 2014;15(5):496–503. Epub 2014/02/14. 10.4161/cbt.28020 ; PubMed Central PMCID: PMCPmc4026071. 24521660 PMC4026071

[17] Riethdorf S, Muller V, Zhang L, Rau T, Loibl S, Komor M, et al Detection and HER2 expression of circulating tumor cells: prospective monitoring in breast cancer patients treated in the neoadjuvant GeparQuattro trial. Clinical cancer research: an official journal of the American Association for Cancer Research. 2010;16(9):2634–45. Epub 2010/04/22. 10.1158/1078-0432.ccr-09-2042 . 20406831

[18] Gasch C, Bauernhofer T, Pichler M, Langer-Freitag S, Reeh M, Seifert AM, et al Heterogeneity of epidermal growth factor receptor status and mutations of KRAS/PIK3CA in circulating tumor cells of patients with colorectal cancer. Clinical chemistry. 2013;59(1):252–60. Epub 2012/11/09. 10.1373/clinchem.2012.188557 . 23136247

[19] Joosse SA. 2015. Available from: http://in-silico.net/tools/statistics/fisher_exact_test

[20] Joosse SA. 2015. Available from: http://in-silico.net/tools/statistics/survivor

[21] Cohen SJ, Punt CJ, Iannotti N, Saidman BH, Sabbath KD, Gabrail NY, et al Prognostic significance of circulating tumor cells in patients with metastatic colorectal cancer. Ann Oncol. 2009;20(7):1223–9. Epub 2009/03/14. mdn786 [pii] 10.1093/annonc/mdn786 . 19282466

[22] Huang SC, Lin JK, Lin TC, Chen WS, Yang SH, Wang HS, et al Concordance of Carcinoembryonic Antigen Ratio and Response Evaluation Criteria in Solid Tumors as Prognostic Surrogate Indicators of Metastatic Colorectal Cancer Patients Treated with Chemotherapy. Ann Surg Oncol. 2015;22(7):2262–8. Epub 2015/01/15. 10.1245/s10434-014-4228-y . 25586242

[23] Strimpakos AS, Cunningham D, Mikropoulos C, Petkar I, Barbachano Y, Chau I. The impact of carcinoembryonic antigen flare in patients with advanced colorectal cancer receiving first-line chemotherapy. Ann Oncol. 2010;21(5):1013–9. Epub 2009/10/29. mdp449 [pii] 10.1093/annonc/mdp449 . 19861580

[24] Iwanicki-Caron I, Di Fiore F, Roque I, Astruc E, Stetiu M, Duclos A, et al Usefulness of the serum carcinoembryonic antigen kinetic for chemotherapy monitoring in patients with unresectable metastasis of colorectal cancer. J Clin Oncol. 2008;26(22):3681–6. Epub 2008/08/02. 26/22/3681 [pii] 10.1200/JCO.2007.15.0904 . 18669452

[25] Aggarwal C, Meropol NJ, Punt CJ, Iannotti N, Saidman BH, Sabbath KD, et al Relationship among circulating tumor cells, CEA and overall survival in patients with metastatic colorectal cancer. Ann Oncol. 2013;24(2):420–8. Epub 2012/10/03. mds336 [pii] 10.1093/annonc/mds336 . 23028040

[26] Bidard FC, Peeters DJ, Fehm T, Nole F, Gisbert-Criado R, Mavroudis D, et al Clinical validity of circulating tumour cells in patients with metastatic breast cancer: a pooled analysis of individual patient data. The Lancet Oncology. 2014;15(4):406–14. Epub 2014/03/19. 10.1016/s1470-2045(14)70069-5 . 24636208

[27] Hou JM, Krebs MG, Lancashire L, Sloane R, Backen A, Swain RK, et al Clinical significance and molecular characteristics of circulating tumor cells and circulating tumor microemboli in patients with small-cell lung cancer. Journal of clinical oncology: official journal of the American Society of Clinical Oncology. 2012;30(5):525–32. Epub 2012/01/19. 10.1200/jco.2010.33.3716 . 22253462

[28] Musella V, Pietrantonio F, Di Buduo E, Iacovelli R, Martinetti A, Sottotetti E, et al Circulating tumor cells as a longitudinal biomarker in patients with advanced chemorefractory, RAS-BRAF wild-type colorectal cancer receiving cetuximab or panitumumab. International journal of cancer Journal international du cancer. 2015;137(6):1467–74. Epub 2015/02/24. 10.1002/ijc.29493 . 25704501

[29] Andreopoulou E, Yang LY, Rangel KM, Reuben JM, Hsu L, Krishnamurthy S, et al Comparison of assay methods for detection of circulating tumor cells in metastatic breast cancer: AdnaGen AdnaTest BreastCancer Select/Detect versus Veridex CellSearch system. International journal of cancer Journal international du cancer. 2012;130(7):1590–7. Epub 2011/04/07. 10.1002/ijc.26111 . 21469140

[30] Van der Auwera I, Peeters D, Benoy IH, Elst HJ, Van Laere SJ, Prove A, et al Circulating tumour cell detection: a direct comparison between the CellSearch System, the AdnaTest and CK-19/mammaglobin RT-PCR in patients with metastatic breast cancer. British journal of cancer. 2010;102(2):276–84. Epub 2009/12/03. 10.1038/sj.bjc.6605472 ; PubMed Central PMCID: PMCPmc2816650. 19953098 PMC2816650

[31] Heitzer E, Auer M, Gasch C, Pichler M, Ulz P, Hoffmann EM, et al Complex tumor genomes inferred from single circulating tumor cells by array-CGH and next-generation sequencing. Cancer research. 2013;73(10):2965–75. Epub 2013/03/09. 10.1158/0008-5472.can-12-4140 . 23471846

[32] Gorges TM, Tinhofer I, Drosch M, Rose L, Zollner TM, Krahn T, et al Circulating tumour cells escape from EpCAM-based detection due to epithelial-to-mesenchymal transition. BMC cancer. 2012;12:178 Epub 2012/05/18. 10.1186/1471-2407-12-178 ; PubMed Central PMCID: PMCPmc3502112. 22591372 PMC3502112

[33] Ueo H, Sugimachi K, Gorges TM, Bartkowiak K, Yokobori T, Muller V, et al Circulating tumour cell-derived plastin3 is a novel marker for predicting long-term prognosis in patients with breast cancer. British journal of cancer. 2015;112(9):1519–26. Epub 2015/04/17. 10.1038/bjc.2015.132 ; PubMed Central PMCID: PMCPmc4453677. 25880010 PMC4453677

[34] Yokobori T, Iinuma H, Shimamura T, Imoto S, Sugimachi K, Ishii H, et al Plastin3 is a novel marker for circulating tumor cells undergoing the epithelial-mesenchymal transition and is associated with colorectal cancer prognosis. Cancer research. 2013;73(7):2059–69. Epub 2013/02/05. 10.1158/0008-5472.can-12-0326 . 23378342

[35] Ye X, Weinberg RA. Epithelial-Mesenchymal Plasticity: A Central Regulator of Cancer Progression. Trends in cell biology. 2015;25(11):675–86. Epub 2015/10/07. 10.1016/j.tcb.2015.07.012 ; PubMed Central PMCID: PMCPmc4628843. 26437589 PMC4628843

[36] Huang RY, Guilford P, Thiery JP. Early events in cell adhesion and polarity during epithelial-mesenchymal transition. Journal of cell science. 2012;125(Pt 19):4417–22. Epub 2012/11/21. 10.1242/jcs.099697 . 23165231

[37] Armstrong AJ, Marengo MS, Oltean S, Kemeny G, Bitting RL, Turnbull JD, et al Circulating tumor cells from patients with advanced prostate and breast cancer display both epithelial and mesenchymal markers. Molecular cancer research: MCR. 2011;9(8):997–1007. Epub 2011/06/15. 10.1158/1541-7786.mcr-10-0490 ; PubMed Central PMCID: PMCPmc3157566. 21665936 PMC3157566

[38] Kasimir-Bauer S, Hoffmann O, Wallwiener D, Kimmig R, Fehm T. Expression of stem cell and epithelial-mesenchymal transition markers in primary breast cancer patients with circulating tumor cells. Breast cancer research: BCR. 2012;14(1):R15 Epub 2012/01/24. 10.1186/bcr3099 ; PubMed Central PMCID: PMCPmc3496132. 22264265 PMC3496132

[39] Schwarzenbach H, Pantel K. Circulating DNA as biomarker in breast cancer. Breast cancer research: BCR. 2015;17(1):136 Epub 2015/10/11. 10.1186/s13058-015-0645-5 ; PubMed Central PMCID: PMCPmc4599311. 26453190 PMC4599311

[40] Bettegowda C, Sausen M, Leary RJ, Kinde I, Wang Y, Agrawal N, et al Detection of circulating tumor DNA in early- and late-stage human malignancies. Science translational medicine. 2014;6(224):224ra24 Epub 2014/02/21. 10.1126/scitranslmed.3007094 ; PubMed Central PMCID: PMCPmc4017867. 24553385 PMC4017867

[41] Diehl F, Schmidt K, Choti MA, Romans K, Goodman S, Li M, et al Circulating mutant DNA to assess tumor dynamics. Nature medicine. 2008;14(9):985–90. Epub 2008/08/02. 10.1038/nm.1789 ; PubMed Central PMCID: PMCPmc2820391. 18670422 PMC2820391

[42] Siravegna G, Mussolin B, Buscarino M, Corti G, Cassingena A, Crisafulli G, et al Clonal evolution and resistance to EGFR blockade in the blood of colorectal cancer patients. Nature medicine. 2015;21(7):795–801. Epub 2015/06/02. 10.1038/nm.3870 . 26030179 PMC4868598

[43] Tie J, Kinde I, Wang Y, Wong HL, Roebert J, Christie M, et al Circulating tumor DNA as an early marker of therapeutic response in patients with metastatic colorectal cancer. Annals of oncology: official journal of the European Society for Medical Oncology / ESMO. 2015;26(8):1715–22. Epub 2015/04/09. 10.1093/annonc/mdv177 ; PubMed Central PMCID: PMCPmc4511218. 25851626 PMC4511218

[44] Schwarzenbach H, Machado da Silva A, Calin G, Pantel K. Data Normalization Strategies for MicroRNA Quantification. Clinical chemistry. 2015 Epub 2015/09/27. 10.1373/clinchem.2015.239459 . 26408530 PMC4890630

